# ESBL-plasmids carrying toxin-antitoxin systems can be “cured” of wild-type *Escherichia coli* using a heat technique

**DOI:** 10.1186/1757-4749-5-34

**Published:** 2013-11-19

**Authors:** Katharina Schaufler, Lothar H Wieler, Torsten Semmler, Christa Ewers, Sebastian Guenther

**Affiliations:** 1 Centre for Infection Medicine, Institute of Microbiology and Epizootics, Freie Universität Berlin, Robert von Ostertag-Str. 7-13, Berlin 14136, Germany; 2 Institute of Hygiene and Infectious Diseases of Animals, Justus-Liebig-Universität Giessen, Frankfurter Str. 85-89, Giessen 35392, Germany

**Keywords:** Plasmid, ESBL, *E. coli*, Toxin-antitoxin system, Plasmid-“cured”-variant

## Abstract

**Background:**

Plasmid-encoded extended-spectrum beta-lactamase (ESBL)-enzymes are frequently produced by *Escherichia coli.* Several ESBL-plasmids contain genes for toxin-antitoxin (TA) systems, which assure the maintenance of plasmids in bacteria and prevent the cells from “post-segregational killing”. These systems limit options to “cure” plasmids of ESBL-wild-type strains due to the death of the bacterial cells. A helpful tool to understand the role of ESBL-plasmids in the dissemination of pandemic multi-resistant *E. coli* are ESBL-plasmid-“cured”-variants (PCVs) and their comparison to ESBL-wild-type strains. The purpose of this study was to construct PCVs of ESBL-wild-type *E. coli* strains despite the presence of genes for TA systems.

**Findings:**

Using enhanced temperatures and brain-heart-infusion broth it was possible to construct viable PCVs of wild-type ESBL-*E. coli* strains. The occurrence of TA system-genes including *hok/sok, srnB/C, vagC/D, pemI/K* on ESBL-plasmids of replicon types FIA or FIB was demonstrated by bioinformatic analyses. The loss of the plasmid and the genetic identity of PCV and corresponding wild-type strain was confirmed via different methods including plasmid-profile-analysis, pulsed-field gel electrophoresis and bioinformatics using generated whole genome data of the strains.

**Conclusions:**

This short report describes the successful construction of viable PCVs of ESBL-wild-type *E. coli* strains. The results are hence surprising due to the fact that all “cured” ESBL-plasmids contained at least one complete toxin-antitoxin system, whose loss would normally mean the death of bacterial cells.

## Introduction

Pathogenic *Escherichia coli* cause a wide range of infectious diseases in various animal species and humans, including diarrhoea [1], meningitis, urinary tract and, soft tissue infections [2]. Many *E. coli* produce extended-spectrum beta-lactamase (ESBL)-enzymes, which- in addition to penicillins and others- hydrolyse newer, third-generation cephalosporins and monobactams [3], and limit antimicrobial therapy. Several ESBL-gene families (*bla*_CTX-M_, *bla*_SHV_, *bla*_TEM_ and *bla*_OXA_) are encoded on plasmids [4]. Prior research has demonstrated that some of the ESBL-carrying plasmids influence factors other than resistance, like the ability of *Klebsiella* strains to invade epithelial cells [5]. In these ESBL-plasmids, addiction models such as toxin-antitoxin (TA) systems have been described [6], which prevent the cell from “post-segregational killing”, therefore ensuring the maintenance of the plasmid in the bacterial cell during replication [7]. Several TA systems have been discovered in Gram-negative and Gram-positive bacteria differing basically in functionality and type of the antitoxin. Type I system antitoxins are small antisense RNA molecules, which mostly inhibit toxin mRNA translation or degrade toxin mRNA while type II system protein antitoxins interact post-translationally with protein toxins. Other systems are type III to type V TA systems [8-10]. One of the first [11] and most studied is type I *hok/sok* TA system in *E. coli*. Following replication, in plasmid-free daughter cells type I unstable RNA antitoxin molecules (e.g. sok) degrade rapidly, while stable toxins (e.g. hok) induce cell membrane porins, therefore impairing ATP synthesis and subsequently causing bacterial cell death [12]. In ESBL-plasmids most frequently represented systems not only include *hok/sok* but also *pemK/I* and *ccdA/B*, which seem to be associated with CTX-M-15 and CTX-M-9 encoding plasmids of IncF replicon type [13]. To investigate, which important role ESBL-plasmids play concerning both resistance and factors not related to resistance it is necessary to construct ESBL-plasmid-“cured”-variants (PCVs) and compare those pheno- and genotypically to ESBL-wild-type strains. Different methods are known to “cure” bacterial plasmids, most of them use chemical treatment like ethidium bromide or acridine orange in different concentrations added to bacteria in Luria-Broth (LB) [14]. These methods often involve the problem that “curing” the ESBL-plasmid of the wild-type strain causes the death of the bacterium due to the loss of an operating TA system. In this study we “cured” TA systems-containing ESBL-plasmids from wild-type *E. coli* testing an established method [15] using enhanced temperatures, brain-heart-infusion (BHI) medium and several weeks of continuing sub-cultivation protocols.

## Material and methods

To force the loss of the ESBL-plasmid, a heat technique was performed [15]. Single colonies of seven wild-type ESBL-*E. coli* strains (VB977549, IMT19205, IMT27685, IMT16316, VB964041.2, IMT21183, IMT23463 of successful and pandemic sequence types [STs] ST131 and ST648 and different hosts including humans, companion and wild animals (Table 1)) were picked and inoculated in 5 ml BHI broth. BHI tubes were incubated at 45°C for 24 hours. Ten microliters of the overnight culture were spread on CHROMagar^TM^ plates. Plates were incubated at 37°C overnight. Replicate CHROMagar^TM^ plates containing an identical numbered grid on the backside of the plate were then prepared. One contained cefotaxime (4 μg/ml cefotaxime) and the other was prepared without supplementation of antibiotics. Twenty single colonies of each strain were randomly picked from the overnight incubated CHROMagar® plate and single colonies were placed on their identical grid locations in the agar of the replicate plates. This was to ensure that colonies from the two different plates could be assigned to the previously selected, single colony. ESBL-plasmid-“cured” single clones should not grow on cefotaxime-containing plates. These visually “cured” single clones were picked from the corresponding CHROMagar^TM^ plate without cefotaxime according to the grid and their phenotypic resistance against cefotaxime and other antimicrobial classes (Table 2) was screened using agar disc diffusion according to the CLSI method [16]. They were further investigated using plasmid-profile-analysis to prove the loss of the plasmid [17]. Clonal identity of the wild-type and the ESBL-plasmid-“cured”-variant was tested via *Xba*I-pulsed-field gel electrophoresis (PFGE) [18] and following comparative bioinformatic analyses. First, the number of orthologous genes in a pairwise comparison of the genome of the wild-type strain and the corresponding plasmid-“cured”-variant was checked using the OrthoMCL pipeline [19]. In a second approach the phylogenetic distances of all strains were tested. The set of genes, which is present in each of all strains, the Maximum Common Genome (MCG), was therefore calculated, the allelic variants of the MCG from the strains was then extracted and a multiple alignment was built (Semmler, personal communication). Verified ESBL-plasmid-free strains were henceforward named PCV (plasmid-“cured” variant: PCV977549, PCV19205, PCV27685, PCV16316, PCV964041.2, PCV21183 and PCV23463). Presence of genes for TA systems on plasmids was investigated evaluating sequence data using bioinformatic methods. Both wild-type strains as well as PCVs were sequenced by an Illumina HiSeq 2000 sequencer. The resulting reads for the PCVs were used for a *de novo* assembly (CLC Genomics Workbench 6.5, CLC Bio, Denmark). The contigs were then used as reference sequences for a reference mapping of the reads from the wild-type strains. All reads from the wild-type strains, which could not be mapped to the PCV sequence are supposed to represent the extracted plasmids and were used for another *de novo* assembly, which resulted in the contigs of the plasmid sequences. Using BLAST for the plasmid and the PCV contigs, the genes for TA systems could be localized. Other plasmid-“curing” methods including treatment of bacteria with acridine orange and ethidium bromide [14] were additionally tested and modified, however, as they turned out not to be successful, data are not included in this manuscript.

**Table 1:** Origin and genotypical characteristics of the ESBL-wild-type strains

**Strain designation**	**Host**	**Origin**	**Sequence-type**	**ESBL-type**	**Plasmid-replicon- type**	**Toxin-antitoxin system**	**Other epigenomic resistance genes**
VB977549	Dog *(C. lupus familiaris)*	Urinary tract infection	131	CTX-M-14, CTX-M-15	FIA/FIB	*pemI/K, vagC/D, hok/sok*	*bla*_TEM-1_, *bla*_OXA-1_, *tet*(A), *tet*(R)*, aadA, aac(6’)-ib-cr*
IMT19205	Brown rat *(R. norvegicus)*	Feces	131	CTX-M-9a, CTX-M-14, CTX-M-15	FIA/FIB	*hok/sok*	*bla*_TEM-1_, *tet*(A), *sul2, strA, aac(3)-IV, aac(6’)-Ib-cr.*
IMT27685	Raven *(C. corax)*	Feces	131	CTX-M-15	not typed	*pemI/pemK, vagC/D*	*bla*_OXA-1_*, tet*(A), *sul1, strA, strB aac(6’)-Ib-cr*
IMT16316	Blackbird *(T. merula)*	Feces	648	CTX-M-14, CTX-M-15	FIA/FIB	*pemI/K, vagC/D, srnB/C*	*tet*(A), *tet*(R)*, sul1, sul2, strA, strB, aadA, aac(3)-II mph(A), mrx, mphR, dhfrVII,*
VB964041.2	Horse *(E. ferus caballus)*	Soft tissue/wound infection	648	CTX-M-15	FIA/FIB	*pemI/K, vagC/D, srnB/C*	*tet*(A), *tet*(R)*, sul1, sul2, strA, strB, aadA, mph(A), mphR, dhfrVII*
IMT21183	Human *(H. sapiens)*	Urinary tract infection	648	CTX-M-14, CTX-M-15	FIA	*vagC/D, srnB/C*	*tet*(A), *tet*(R)*, sul1, sul2, strA, strB, aadA, aac(3)-II, mph(A), mphR, dhfrVII*
IMT23463	Monk vulture *(A. monachus)*	Feces	648	CTX-M-9	FIB	*PemI/pemK, srnB/C, hok/sok*	*bla*_TEM-1_, *bla*_OXA-1_, *tet*(A),*sul2, strA, strB, aac(6’)-Ib-cr*

**Table 2:** Results of agar disc diffusion testing

**Strain designation**	**Cefotaxime**	**Chloramphenicol**	**Enrofloxacin**	**Gentamicin**	**Streptomycin**	**Tetracycline**	**Sulfonamid-trimethoprim**
VB977549	R	S	R	S	R	R	S
PCV977549	S	S	R	S	S	S	S
IMT19205	R	S	R	R	I	S	S
PCV19205	S	S	S	R	S	S	S
IMT27685	R	R	R	S	R	R	R
PCV27685	S	S	R	S	S	S	S
IMT16316	R	S	R	R	R	R	R
PCV16316	S	S	R	S	S	S	S
VB964041.2	R	S	R	R	R	R	R
PCV964041.2	S	S	R	S	S	S	S
IMT21183	R	S	R	R	R	R	R
PCV21183	S	S	R	S	S	S	S
IMT23463	R	S	R	S	R	R	R
PCV23463	S	S	R	S	R	R	R

(R = resistant, S = sensitive, I = intermediate).

## Results

**Figure 1:**
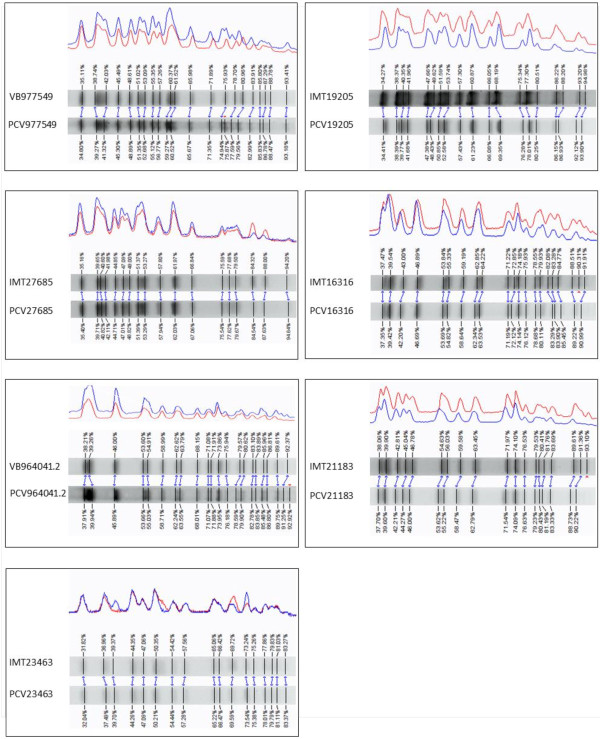
**Dendrograms of the pulsed-field gel electrophoresis.** Wild-type ESBL*-E. coli* and PCV strains after macrorestriction with *Xba*I and PFGE (dice similarity value >98% for all strains). Software: Bionumerics (Applied Maths, Belgium).

**Figure 2:**
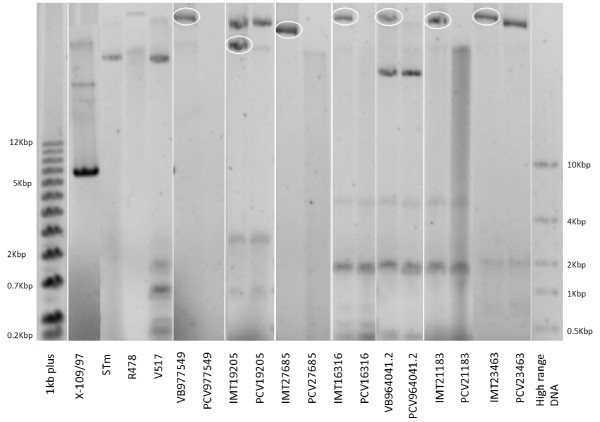
**Electropherogram of the plasmid-profile-analysis.** Wild-type (IMT/VB, left) and ESBL-plasmid-“cured” variants (PCVs, right) illustrated in pairs. Revolved in white are the big plasmids in the ESBL-wild-type strains, which have been “cured” in the corresponding PCVs. At the beginning and the end are several markers (1 kb plus [Thermo Scientific, USA], X-109/97 [12,5MDa], STm [60MDa], R478 [166MDa], V517 [36.8/4.8/3.7/2.0/1.8/1.4], FastRuler High range DNA [ThermoScientific USA]).

Treatment of bacteria with enhanced temperatures was performed to construct viable toxin-antitoxin system-containing ESBL-plasmid-“cured”-variants of wild-type ESBL-*E. coli* strains. Following three to six weeks of daily sub-cultivation, examination of the phenotypical resistance status of the wild-type ESBL-strains revealed seven cefotaxime-susceptible and phenotypically ESBL-plasmid-“cured”-variants. Table 2 shows the results of agar disc diffusion testing of cefotaxime and other classes of antimicrobials. All PCVs lost their cefotaxime resistance along with most of the non-beta lactam resistances. Clonal identity of the PCV and its wild-type strain was proven via pulsed-field gel electrophoresis (Figure 1) and bioinformatic analyses. The seven PCV strains showed a highly similar macrorestriction pattern compared to their corresponding wild-type strain. For most of the PCV strains, a small band was missing in the patterns, which might be the “cured” plasmid itself (PCV977549, PCV16316, PCV964041.2 and PCV21183). Comparing orthologous genes, similar results were obtained for all pairs, which showed only one significant excess of genes in the wild-type strain without an ortholog in the “cured”-variant in an amount that corresponds to the size of the plasmid. In case of phylogenetic distances a clustering confirmed the genetic identity for each pair of wild-type and plasmid-“cured”-variant strain (data not shown). The loss of large plasmids (>100 kb) was approved via plasmid-profile-analysis (Figure 2). Plasmid-profile analysis revealed no loss of other smaller plasmids apart from the large ESBL-resistance plasmids. Bioinformatic analyses confirmed the presence of different TA system-genes on plasmids including *hok/sok, srnB/C, vagC/D, pemI/K.* All but one wild-type strain (IMT19205) carried ESBL-plasmids encoding for multiple TA systems, whereat all plasmids belonged either to replicon types FIA or FIB (Table 1).

## Conclusions and discussion

As ESBL-plasmid-“curing” of the strains applied in this study did not work treating bacteria with ethidium bromide or acridine orange a previously described method [15] using a heat technique was performed. This method was adequate to construct viable PCVs and to our knowledge we are the first ones to describe a successful “curing” of ESBL-plasmids, which carry genes for toxin-antitoxin systems. The results are surprising because one might think that bacteria cannot survive without this plasmid. Strains used in this study all had at least one complete ESBL-plasmid-encoded toxin-antitoxin system, including *hok/sok, srnB/C, vagC/D* and *pemI/K,* which were encoded on plasmids of replicon types FIA or FIB carrying *bla*_CTX-M-9_, *bla*_CTX-M-14_ and *bla*_CTX-M-15_ or combinations of these three ESBL-genes. The addiction systems and replicon types found have been characterized in ESBL-producing *E. coli* before and certain combinations of ESBL-enzyme, addiction systems and replicon type might contribute to the success and spread of multi-resistant *E. coli* strains [13]. An association of CTX-M-15 with FIA and CTX-M-9 with FIB plasmids has been described before and the same was true for the isolates used in this study (Table 1). Mnif *et al.* also observed that the occurrence of CTX-M-14 correlated with FII replicons, however, FIB replicon type plasmids carrying CTX-M-14 have also been found in this earlier study [13]. No correlation for CTX-M-14 and FII replicons was observed in this study, as this ESBL-type was also present on FIA/FIB plasmids (Table 1). This might be partly due to the fact that *bla*_CTX-M-14_ was only detected in combination with other CTX-M enzymes (Table 1). So why did bacteria, whose TA system-containing plasmid was “cured” using a heat technique, survive? One might speculate that some toxins degenerate irreversibly above certain temperatures or that the TA genes are not expressed at 45°C. Or is it that continuing soft stimulation using high temperatures allows a slow adaption to changing environmental conditions and therefore “curing” of the plasmid, while chemicals, like ethidium bromide, together with the loss of a TA system mean too much stress for bacteria? Pulsed-field gel electrophoresis, plasmid-profile-analysis and bioinformatic analyses confirmed that the genetic change is restricted solely to the loss of an ESBL-plasmid and that genetic identity of PCV and wild-type strain remains given. Constructed PCV strains might be important tools to investigate the influence of an ESBL-plasmid on its bacterial host.

## Competing interests

The authors declare that they have no competing interests.

## Authors’ contribution

KS designed and performed the experiments, structured and prepared the manuscript. CE and LHW drafted and revised the manuscript critically for important intellectual content and took part in writing of the manuscript. TS performed bioinformatic analyses. SG participated in the design of the study, revised the manuscript critically for important intellectual content and took part in writing. All authors read and approved the final manuscript.
